# Where Do, and Should, Science and Science Education Happen?

**DOI:** 10.1007/s11191-025-00651-2

**Published:** 2025-06-02

**Authors:** Cyrus C. M. Mody

**Affiliations:** Faculty of Arts and Social Sciences, Maastricht University, Maastricht, Netherlands

**Keywords:** History of science, Scientific practice, Hybrid science, Nature of Science

## Abstract

Wherever scientific research can be found, science education is usually present too, and vice versa. Moreover, the sites of science and science education often contain much else besides: commerce, bureaucracy, spiritual exploration, family relationships, national security, occupational/environmental health and safety, activism, and so on. Even practices that are clearly scientific overlap, borrow, and resemble practices associated with other domains: art, music, cookery, reading, writing, business, and more. This article illustrates the hybridity of science by reviewing the history of science literature and offering examples from the author’s past work. That hybridity should inform any science education pedagogy that gestures to the “Nature of Science” and/or that centers on scientific practice and not just formal scientific knowledge. Practices resembling scientific practice can be found in many parts of the non-science curriculum and could be taught in the science classroom and/or in other classrooms.

## Introduction

The articles in this special issue ask whether exchanges between history of science and science education can be revitalized and updated in light of the evolution of both fields over the past several decades. In particular, the aim of the special issue is to take account of both fields’ (so far mostly separate) attempts to provincialize Euroamerican science and science education. That aim hinges in part on matters of space and place. Thanks largely to the legacy of colonialism, formal science education almost everywhere tends to privilege scientific knowledge created in the Global North over knowledge created elsewhere. The national disparities in numbers of Nobel laureates (von der Heyden, 2024), for instance, contribute to science textbooks that are filled with laws and concepts named after Euroamericans, with very few named after scientists from the Global South. For related reasons, *science* everywhere tends to privilege certain sites of science *education*. Think, for example, of the dominance of holders of degrees from the US Ivy Plus schools in disciplines such as economics (Albarrán et al., 2017). Yet, history shows that, in fact, people from all places and trained at all kinds of institutions are capable of creating robust scientific knowledge, even if power asymmetries sustain and memorialize contributions from the Global North more than those from the Global South (Prasad, 2014).

This essay is less focused on geographic or cultural disparities in science and science education than the rest of the special issue. Instead, I proceed from the contention that those disparities are based on an idealized and stylized conception of what science and science education are (McComas, 1998; Allchin, 2013). If what gets taught as “science” is only the outcome of interrogating Nature (Merchant, 1980) ever more intrusively—with more and more expensive equipment (Hallonsten, 2016), in ever more expensive laboratories, operated by ever-expanding teams of specialists with expensive educations from select universities—then of course “science” will be associated more with wealthy countries that can more easily afford those things. As I will argue, however, historians of science have abundantly shown that scientific knowledge-making is a much more hybrid activity than the stylized picture allows. Even in rich countries, science includes much that is economical, *ad hoc*, and embedded in resource-poor settings; thus, at least some aspects of science can be accomplished in rich and poor countries alike (Sekhsaria, 2016; Werrett, 2021). Moreover, much of science resembles activities common in other vocations and avocations, and thus at least some aspects of science can be accomplished, and certainly evaluated rigorously, by non-professionals, whether in the Global North or South (Ottinger, 2020). Science education, too, is more widely dispersed than the iron grip of elite schools would indicate. Even elite scientists learn many of science’s essential skills outside of their *alma mater*, in ordinary locales that are open to anyone. The roads to membership in science’s elite—or even roads that bypass such an elite entirely—therefore need not run so disproportionately through a handful of institutions with high barriers to entry.

This essay proceeds in four parts. First, I will concretely illustrate the hybrid and dispersed character of science and science education by sketching the biographies of two physicists, Virgil Elings and David Phillips, about whom I’ve written extensively elsewhere (Mody, 2011; Mody, 2022). Then, I will survey what other historians of science have said about the hybrid and dispersed character of science and science education: first by identifying some unexpected answers to the question “where do science and science education happen?” and secondly by identifying some unexpected other things—neither science nor science education—that also happen in those same places. Next, I compare and connect historians’ picture of a hybrid, dispersed science to that offered by the “Nature of Science” program in science education. Finally, I will offer my own speculative suggestion for how science educators might put the *many* places of science and science education in better contact with each other.

## Virgil Elings and David Phillips

To summarize: my argument is twofold:

1. Science and science education happen in many places, not just the laboratory or the classroom. Therefore, *some* form of good science and science education can be done by all people in all parts of the world and not just by those with access to the most expensive, well-equipped laboratories and classrooms.
2. Wherever science and science education happen, including in the laboratory and classroom, other activities are also simultaneously going on. Those other activities resemble, overlap, and borrow from walks of life not conventionally associated with science or education. Therefore, non-scientists potentially can contribute to and evaluate aspects of science and science education that relate to their own lifeworld. Conversely, participating in science and science education can aid all people in their chosen occupations, not just (future) scientists.

To help readers grasp that abstract argument more concretely, I now offer a profile of two physicists, Virgil Elings and David Phillips, whose careers thoroughly exemplify the hybrid, dispersed character of science and science education. I have written two books in which Elings figures prominently, the second of which also features Phillips. The source base for those books was a combination of ethnographic fieldwork, oral history interviews, archival documents, and published work. So as not to overburden this essay with references to unpublished sources, I direct readers to Mody (2011, chapters 4 through 6) and Mody (2022, chapter 2); here, I will mostly cite relevant *published* sources.

Elings and Phillips were not Nobel prize winners, there are no scientific laws named after them, and their published output was relatively modest. They were accomplished working scientists, not academic stars (though Elings was well-known in certain research communities). Yet, because of that ordinariness, their biographies are illustrative of broader trends in postwar USA—and to some extent global—science *and* science education.

Both were born in Iowa around 1940 and graduated from Iowa State University in the early 1960s (though they apparently did not know each other at that point) as part of the generation of US students recruited into science and engineering fields in the wake of *Sputnik* (Lucena, 2005). They both then pursued PhDs in prestigious physics programs: Elings at MIT, Phillips at the University of California, Berkeley. Notably, Elings was at that point primarily a high-energy physicist, and his dissertation research therefore placed him at the scene of one of the more shocking episodes in Cold War academic physics research: the 1965 explosion at the Cambridge Electron Accelerator (CEA) jointly run by Harvard and MIT, in which a 19-year-old technician died and several other people were badly injured (Anonymous, 1965; Chadis, 2004; Langer, 1965). As Peter Galison (1997) has shown, that explosion had significant consequences for collider physics worldwide. I would add that any facility where graduate students and teenage technicians are working is likely to feature considerable learning on the job, while any site housing hundreds of gallons of liquid hydrogen *should* feature rigorous safety practices and emergency preparedness—all of which seemingly came up short in the case of the CEA.

During or just after their graduate studies, Phillips and Elings did short stints in the national security sector: Phillips (1966) at the Naval Research Laboratory and Elings with Union Carbide’s Nuclear Division and then with Autonetics, a division of North American Aviation. Both were then hired into tenure-track positions at the University of California, Santa Barbara (UCSB) in 1966. At UCSB, both were expected to help their department improve its reputation for cutting-edge research so that it could attract more PhD students, more prominent and talented faculty members, and more federal grants. That strategy eventually worked for the department—now one of the leading physics departments in the world—but not for Elings and Phillips individually. Both struggled to attract external funding and especially PhD students—the lifeblood of any postwar US physics department looking to move up in the rankings. One of Phillips' very few publications from those years (Phillips & West, 1970), for instance, was a description in the *American Journal of Physics*—a periodical primarily by and for teachers of undergraduate physics courses—of a “poor man’s nitrogen laser” for teaching rather than for research.

Phillips was also involved in efforts at public outreach and citizen science, in part by helping a technician in his department, Tony Korda, to establish a public-facing Physics Learning Center. More controversially, Phillips served as the research director for the Southern California Society for Psychical Research, an organization that included a few academics but also many members not affiliated with universities. In 1973, Phillips even brought the famous Dutch “psychic detective” Gerard Croiset into a UCSB lab to test Croiset’s psychokinetic abilities on one of his colleagues’ experimental apparatus.^1^ From the perspective of his departmental seniors, then, Phillips had hybridized science with pseudoscience. Phillips, of course, believed that parapsychology was, or at least could be turned into, a science. My own view is that whether parapsychology is a science or not, Phillips’ career shows that parapsychology certainly encompasses research that can be done both in well-equipped university labs and at more unusual locations such as the Esalen countercultural retreat or the experimenter’s own home (Collins & Pinch, 1982; Kaiser, 2012). Also, like many sciences *and* non-scientific activities, parapsychology contains a strong element of spiritual exploration (Stanley, 2007) and individual self-actualization (Bardini, 2000).

In any case, it is perhaps unsurprising that Phillips was denied tenure and Elings’ promotion to associate professor was long delayed. Elings’ position became somewhat more secure than Phillips’ in 1971, when he was made director of the UCSB Physics department’s new Master of Scientific Instrumentation (MSI) program that brought in paying students at a time when both PhD and undergraduate enrollments in physics were falling due to fiscal and political fallout from the Vietnam War (Leslie, 1993). Phillips (1971), meanwhile, found work with a local start-up, Science Spectrum, where his main job was to adapt an instrument invented by another physicist for use by biologists (and to get biologists to actually buy and use it). He also assisted Elings with the MSI program and taught parapsychology courses in UCSB’s Tutorial Program, a sort of test-bed for alternative pedagogies.

The MSI program also became something of a pedagogical test-bed in the early 1970s. Students pushed for, and Elings and Phillips gladly created, opportunities to learn outside the classroom by building all sorts of instruments for real users. Initially their “customers” were mainly UCSB scientists who could no longer afford to buy laboratory equipment due to federal R&D budget cuts, but eventually they also began developing new instruments for extramural organizations such as a local hospital. Some also took on projects for individual clients, such as a man who needed a stereo system specially modified to suit his visual impairment. Elings’ advertisements for the program trumpeted its interdisciplinary, applied, extramural character, as did student evaluations. He and his co-instructors also talked up the program in the physics trade press (Nicoli et al., 1978; Elings, 1981 & 1995) and physics education journals (Elings & Phillips, 1973), where they particularly highlighted the fact that non-science majors could learn to build instruments quickly and with little classroom instruction.

Meanwhile, both Elings and Phillips were also doing science at home, or rather in their garages. Both had small lifestyle firms—Glendan (Phillips) and Santa Barbara Technology (Elings)—that sold various gadgets broadly related to their other interests. Phillips, for instance, advertised an acupuncture-point locator, the Acupointer, based on Soviet parapsychology research; Elings marketed a similar device, the Tobiscope, which may indicate there was some collaboration between the two. They also sold (and apparently sometimes cross-marketed) devices that were based on MSI student projects, such as an apparatus to help children with speech and hearing disabilities to pronounce vowels properly. Both companies were also sites of family bonding. Phillips’ company was named after his sons, who remembered his garage experiments fondly (Phillips, undated). Elings also involved his sons in his off-campus projects; at least one of them (Jeff Elings) eventually also worked for his more substantial and serious commercial ventures (Anoikin et al., 1998).

Over time, Elings developed a rhythm: take an experimental apparatus invented by one of his students or UCSB collaborators, build a company around it, grow the company until he lost interest, sell the company, and re-invest the proceeds in building a new company. Interestingly, one of his first successes, paving the way for later companies, was a product still sold in science museums all over the world, consisting of two parabolic mirrors with their concave sides facing each other and a small hole in the center of the top mirror; objects placed at the bottom of the lower mirror then appear to float in mid-air. Elings’ big success, though, was with a later company, Digital Instruments (DI), which he co-founded in 1987 with one of his former MSI students (and which employed many other MSI graduates). From the late 1980s to the late 1990s, DI was the world’s leading manufacturer of scanning probe microscopes, both for scientific research and for industrial applications such as quality control.^2^

Frictions with his UCSB colleagues led Elings to leave his faculty position in 1990. However, DI maintained close ties with the university, and especially with another UCSB physics professor, Paul Hansma. Hansma’s students and intellectual property flowed to DI, while DI’s money, equipment, and also intellectual property flowed into Hansma’s lab. Hansma’s instruments tended to be built cheaply and quickly; they were often based on wooden prototypes Hansma constructed in his home woodshop, and they sometimes incorporated parts scavenged from local pawn shops. That is, like Elings’ and Phillips’, Hansma’s science took place partly in the home and other unconventional sites.

As with his earlier ventures, Elings eventually lost his enthusiasm for Digital Instruments and sold the company in 1998. Between the proceeds of that sale and his ex-wife’s considerable fortune from real estate ventures in the Santa Barbara area, Elings and his family have had the means to become big-time philanthropists, giving money to universities (particularly for laboratory buildings), museums, hospitals, parks, high schools, and so on (Chadis, 2004; Conrad, 2007; Keyani, 2009; Leonard, 2006; Welsh, 2016). Phillips never made it as big, but he continued working in the scientific instrumentation start-ups north of the Santa Barbara airport that had been seeded first by Science Spectrum and later by Elings’ various companies and their spin-offs. Today, those companies are seen as the foundation of a thriving industrial cluster that generates millions of dollars in revenue for the university and the State of California. That’s not what Elings or Phillips set out to do, though, and throughout the 1970s and 1980s they faced significant opposition from the university, even if today Elings, in particular, is celebrated as a role model for the kind of scientific entrepreneurs that UCSB now says it wants to train.

## Hybrid Sites of Science and Science Education

Let’s now think through the sites that appeared in Phillips’ and Elings’ joint biography. In all of those sites, science *and* science education were both happening, but so were lots of other things. Obviously, science and science education were both present in the various universities mentioned—though they were often conjoined in unexpected ways. The MSI program, in particular, shows that science can be done in and out of the lab, science education in and out of the classroom. MSI students were doing original scientific research in the course of their projects at organizations all around Santa Barabara—research that some of them published in well-regarded journals, with Elings as co-author (Elings et al., 1977; Elings & Holly, 1973).

The MSI program also shows that anyone has the potential to do good science, no matter their educational pedigree. As Elings put it, “one no longer needs a degree in electrical engineering to design state-of-the-art electronic instruments” (Elings, 1981). Elings was particularly proud of one MSI graduate whose undergraduate major was in psychology and religious studies and who went on to become one of DI’s top designers.

Moreover, the MSI program hybridized much more than just science and science education. Some MSI students were also pursuing a kind of activism by building projects oriented to environmental monitoring, alternative energy, or helping people with visual, hearing, and other disabilities. Some of their projects also became the basis for Elings’ (and to a lesser extent Phillips’) entrepreneurial ventures. Thus, UC Santa Barbara’s classrooms and labs were sites of commerce and activism alongside, indeed mutually implicated with, science and science education. Moreover, Phillips’ and Elings’ pedagogical innovations were grounded in the hybridity and dispersed character of the MSI program; their students were enthusiastic about the program’s experimental character and the opportunities it afforded for them to work at the leading edge of science *because* they learned so much outside of both the classroom and the lab.

The universities mentioned in the biographical narrative above were also sites of activity oriented to national security, occupational health and safety, public engagement, spiritual exploration, and more. Conversely, the companies that appear in Elings’ and Phillips’ biography weren’t just sites of commerce, but also of original scientific research (e.g., DI employees regularly published scientific journal articles) and even education – not just vocational training but at times even a kind of *Bildung*. Elings, in particular, would occasionally convene DI’s employees for inventing sessions, where he would set a design challenge unrelated to the company’s products (one oft-remembered session tasked employees with coming up with a better washing machine). These were, in part, meant to sharpen employees’ design skills; but they also complemented a broader critique—or even a cynical suspicion—of conventional wisdom that Elings systematically cultivated in his students and employees.

Yet the companies in my narrative were also sites of much more than commerce, science, and science education. Some of them were lifestyle firms (Shah & Tripsas, 2007)—essentially hobbyism that pays. Phillips, in particular, used his garage company to earn a little money from his spiritual avocation. Both Phillips’ and Elings’ companies were also sites of family interactions and the passing of values from generation to generation. Finally, the wealth generated by Elings’ companies has further benefited science and science education—not just through his and Betty Elings Wells’ endowment of academic laboratory buildings, but also through their support of science museums and of Dos Pueblos High School’s Engineering Academy (Bascomb, 2011).

The hybridity of all of these sites is rather unsurprising in light of the history of science literature of the past half-century, much of which is devoted to adding to a list of unexpected sites where science happens, and to showing that other things happen at those sites alongside scientific research. Some of those sites are not *so* unexpected, of course, though the heterogeneity of the practices found there might be. Field science, for instance, necessarily takes the scientist outside of the specialized, semi-closed space of the lab. Field disciplines therefore tend to more directly involve Citizen Scientists (Coen, 2013) and non-scientists (Kohler & Vetter, 2016; Raby, 2019; Tabernero, 2022; Vetter, 2019), and to be more obviously engaged with formal and informal education (Kohler, 2002) and with political projects such as colonialism (De Bont, 2021; Pang, 2002), extractivism (Hubbard, 2014; Pannhorst, 2024; Schleper, 2022), and environmentalism (Benson, 2012).

Some field sites—research vessels (Burstyn, 1975), Antarctic stations (Heggie, 2014), spaceships (Karafantis, 2013), isolated “residential” camps (Kohler, 2011)—are also total institutions (Goffman, 1961) from which the researcher cannot escape while they are on expedition, and which therefore require the researcher to recreate not just the lab but also the home, office, restaurant, pub, hospital, etc. Field sites can therefore feature cookery, marital interactions, child-rearing, tourism, and other activities that complement the science and science education found there as well. That is, field sites tend to be open to more kinds of people than expensive laboratories in the Global North; they include practices that non-professional scientists can understand and may even be expert in; they are dispersed all over the world; and while some field sites are resource-intensive, governments, organizations, and individuals in the Global South are more than capable of pursuing field research (Arellano-Escudero, 2021).

Likewise, the state is an obvious site of scientific research alongside its primary function of governance. The state also has a clear interest in education, and globally most schools and universities are public institutions. Yet, both research and education often take place alongside other activities within sites controlled by the state. Perhaps the clearest examples in the history of science and science and technology studies (STS) literatures come from the Manhattan Project and its successor, the US National Laboratories system. During World War II, scientists working at Los Alamos and other remote sites brought their spouses (many of whom also joined the project), their children, even their pianos (Rhodes, 1986; Thorpe & Shapin, 2000). Because the project was secret, newcomers had only rudimentary prior knowledge of what had been accomplished thus far, and so had to be quickly brought up to speed. Thus, the project’s leaders devoted much of their time to pedagogy and pedagogical innovation. After the war, the National Laboratories remained sites of intensive intergenerational learning (McNamara, 2001; Gusterson, 1996). For safety and security reasons, the National Laboratories were also isolated and partially cut off from the world, meaning their researchers pressed for and/or supplied their own on-site amenities such as symphony orchestras and liquor stores—even when doing so led to frictions with the state.^3^

Other hybrid sites of science, science education, and much else would seem rather obvious, and yet have been surprisingly understudied. For instance, even the most cursory inspection reveals that scientists spend much of their time preparing for, journeying to, and performing at conferences—and yet, historical and STS studies of scientific conferences are still relatively few in number and mostly of recent vintage. Even so, that literature has shown that conferences are closely associated with training (Ochs & Jacoby, 1997), learning, and *Bildung*—as well as with diplomacy, tourism, family interactions, gluttony, athletics, conspicuous consumption, and much more (Cherrier & Saïdi, 2021; Somsen, 2023; Kotsou, 2024; Mody, 2017). Scientific discovery and even laboratory research sometimes happen at conferences and on the trains, boats, and airplanes carrying scientists to them (Brown & Lee, 2006).^4^ Likewise, scientific journals are almost by definition sites of science and science education, and yet the historical literature on those institutions is also quite new (Baldwin, 2015; Csiszar, 2018; Fyfe et al., 2022). Nevertheless, that literature has already revealed that journals are hybrid sites of, well, journal-ism, but also of commerce (Noël, 2023), fraud (Reich, 2009), the pursuit of personal quirks and grievances (Zangwill, 2021), and so on.

Then, there are the hybrid sites of science and science education that feature prominently in the historical literature, yet which are consigned to the historical past (if noted at all) in the stylized picture of science conveyed in schools. The home, for instance, was one of the main locales of scientific experimentation—and, of course, of much formal and informal science education—until well into the nineteenth century (Gooday, 1991; Hannaway, 1986; Schaffer, 1998; Shapin, 1988). Its importance certainly receded with science’s professionalization, but never disappeared entirely. Even into the 1940s, the Manhattan Project, again, had its origins partly in the private laboratory—on his estate—of the New York lawyer Alfred Loomis (Conant, 2003). More recently, the 2014 Nobel Prize in Chemistry was awarded partly for the invention of a new kind of microscope, the prototype of which was constructed in the early 2000s in the living room of one of its inventors (Betzig, 2024). Moreover, millions of Citizen Scientists undertake research from their homes and neighborhoods every day (Ottinger, 2020).

Some of those Citizen Scientists belong to virtual communities that closely resemble the “philosophical societies” and “debating clubs” of yore, in the tradition of the Accademia dei Lincei in seventeenth-century Rome or the eighteenth-century Lunar Society of Birmingham. Until the early twentieth century, such clubs remained a powerful force in science, often bringing together professionals and amateurs for intellectual stimulation *and* convivial fraternization in the salon or clubhouse (Alberti, 2001; Bowler, 2009; Shapin & Thackray, 1974). As Lea Beiermann (2023) has shown, the professional *and* amateur members of “postal microscopical societies” of the second half of the nineteenth century also contributed greatly to the commercialization of research, to formal and informal science education, and even to changes to postal regulations that enabled training at a distance—members learned how to operate the microscope from the comfort of their own homes—that resembled today’s online courses.

Religious sites such as monasteries, madrasas, and temple complexes have also long played an important role in both science and science education (Lindberg, 1992). In my experience, though, many students arrive in the classroom believing that such places only ever held back science, and ceased to be relevant with the coming of the Scientific Revolution. The truth is, however, that scientists who were primarily employed as clerics continued to work at the forefront of various scientific fields throughout the twentieth century (Kragh, 2018). Indeed, in places such as Spain under Franco, laboratories and churches were substantially overlapping and entangled places even into the 1970s (Camprubí, 2014).

Finally, there are places where science and science education happen that might be well-known to historians but are probably not seen as sites of scientific work by the general public or even by most science educators. One such is the philanthropic foundation. The main aims of philanthropies, of course, are a mix of charity and improvement of the donors’ reputations, not science. Yet, those foundations that focus on scientific research must decide where to direct their money, i.e., they shape the scientific landscape by building up some fields and not others. Some—particularly the Rockefeller Foundation—have employed prominent scientists such as Warren Weaver, who have used the funds they controlled to actively construct the methods and content of medicine and public health (Brown, 1979), eugenics (Kevles, 1985), agronomy (Fitzgerald, 1986), the life sciences (Abir-Am, 1988; Kay, 1993; Kohler, 1991; Manning, 1983), the social sciences (Solovey, 2013; Crowther-Heyck, 2006), quantum mechanics and nuclear physics ( Assmus, 1993; Krige, 2006), cybernetics (Geoghegan, 2020; Kline, 2015), and so on. Not just the funding of science but science itself—the manufacture of knowledge—goes on within the foundations’ walls.

The same goes for international organizations such as the World Health Organization, International Union for the Conservation of Nature, Food and Agriculture Organization, and so on. There’s now a growing history of science literature on scientists in such organizations (Schleper, 2019; Schmelzer, 2016; Selcer, 2018; Stinsky, 2019), and more generally on scientists as vectors of diplomacy and/or international bureaucracy (Aubin, 2020; Camprubí, 2020; Reinisch, 2023; Zaidi, 2023). Much remains to be said, though, about the hybridity of the sites where scientific diplomacy is carried out—sites such as the home, the mountain or island resort, or the luxury hotel—and the entanglement of international organizations with some of the other forums discussed above, particularly the philanthropy and the club.

The art gallery also turns out to be a place where scientists often pop up, whether driven there by their own interests or drawn there at artists’ invitation (McCray, 2020; Rogers, 2012; Galison & Jones, 1998). The concert hall and recording studio, too, have long offered opportunities for both research and *Bildung* (Bruyninckx, 2018; Hui, 2012; Thompson, 2002). It’s in those kinds of sites—sites that have their own rich pedagogical traditions—that readers can start to glimpse the normative program that I’ll end this essay with. I call that program “the whole curriculum in the science classroom, scientific practice in all the classrooms”—the idea being that if science happens in the art gallery and the concert hall (and art and music happen in the lab), then art and music education can happen in the science classroom and science education can happen in the art and music classrooms.

So, science and science education are dispersed activities—they happen in lots of places, even places not conventionally associated with science or science education. Science/education’s presence in those unconventional places implies that science and science education are also hybrid activities, since they occur alongside other practices that are not usually understood as scientific or educational. Yet, the survey above—and also my sketch of Phillips’ and Elings’ careers—shows that even those sites that are most conventionally associated with mainstream science—the laboratory, the scientific conference, the library, the professor’s office—also feature all sorts of activities that are not usually taught in the science classroom.

After all, as I’ve asked in print elsewhere, what do scientists do all day (Mody, 2015)? What do even credentialed, professional scientists do when they’re at work in places that everyone agrees are sites of scientific practice? If you need some help answering that question, read the previous sections of this essay again for some hints. There, you’ll see that scientists must travel a lot—to field sites, to conferences, to meet with collaborators, or to gain access to specialized pieces of equipment such as particle colliders. At those conferences or when they sit down with their collaborators, they give presentations and ask and answer questions. They read and write: grant proposals, notebook entries, journal articles, peer reviews of journal articles, emails to/from their colleagues. They work in complex organizations, so they must also fill out forms, deal with bureaucracies, sit in meetings, supervise underlings/resist supervision by their bosses. They build and/or buy equipment, materials, software, and other necessities to run their experiments or generate their theories. They eat (Shapin, 1998), they drink (Secord, 1994), they play the violin (Kursell, 2019), they sit in comfortable chairs (Nasim, 2021), some even sleep on their office couches (Kaiser, 2005)—not just to keep their bodies fueled or relax at the end of a long day, but as an integral part of their science. Thus, scientific practice resonates with the practices of tourists, authors and journalists, chefs and food critics, actors and theater critics, bureaucrats, hobbyists, musicians, professional shoppers, and even idle slackers.

## The Hybrid “Nature of Science”

So science is dispersed—it proceeds simultaneously in many interconnected places, not just in the laboratory or other canonical sites. It is also hybrid—wherever science occurs, other things are happening as well. As my survey indicates, these characteristics of science are well-attested by historians of science and STS scholars. Nor are they entirely novel to science education researchers for that matter; in particular, the Nature of Science (NOS) approach draws on “the philosophy, history, sociology, and psychology of science … for guiding science educators in accurately portraying science to students” (McComas et al., 2002).

Reflexive practitioners have at times worried that STS and history of science portrayals of the messiness of science might be pedagogically harmful (Allchin, 2004; Brush, 1974; Leone & Rinaudo, 2020). Yet, a basic contention of the NOS program is that students are engaged (even positively interested!) in learning about science as a varied, living process (i.e., what scientists do) rather than a dead body of facts (i.e., what scientists know—the traditional content of much science education). The NOS program also advocates for having students perform simulations of scientific practices described by historians and sociologists. The rationale here is that students internalize what they’ve learned better when they are active rather than passive learners. Students who have experienced scientific practices for themselves (and/or have been exposed to the history of science) are also better equipped to recognize that science evolves, and thus will be less disoriented when they inevitably encounter new findings that disagree with what they learned before.

Osborne and Allchin (2024) summarize—but also critique—another common justification for NOS: namely, that learning about and emulating scientific practices for adjudicating knowledge allows students to better judge (pseudo)scientific claims for themselves. Osborne and Allchin’s own motivation for NOS is more sociological: not even scientists can know all of the technical content of science, or even a tiny fraction of it, so they have to learn whose knowledge to trust; thus, an NOS-oriented pedagogy can help students to emulate the practices scientists use for gauging which other scientists they can rely on, such that students will learn to identify who the trustworthy scientists are amid a sea of mis- and dis-information.

I mostly agree with the justifications for NOS-based pedagogies listed above. I particularly agree with Osborne and Allchin that Nature of Science has greater potential than traditional science pedagogies for benefiting *all* students, and not just those who will later join the science and engineering workforce. I also find many of the tenets of Nature of Science (McComas, 2002) highly compatible with the history of science literature surveyed above: e.g., that science does *not* have a single, linear, mechanistic “method” for turning hypotheses into theories into laws; that science *is* creative; that science is characterized by debate and even dissent; and that science is a collaborative enterprise.

Yet, in my view, the Nature of Science doesn’t go far enough. The historical examples its proponents cite are salutary, but still mainly mined for their epistemological content (Ryder & Leach, 2008), and the “core NOS ideas” (McComas, 2008) still mainly relate to the cognitive dimensions of science. To be fair, those core ideas do gesture to the distributed (Hutchins, 1995) character of scientific cognition. Yet NOS rather neglects the rich (if also sometimes quite tedious) lived experience of the scientist and the hybrid and dispersed character of science found in the history of science and STS literatures. The NOS program still provides only a handful of canonical answers to the question, “where does science happen?”

That’s especially the case for Nature of Science-based *policy* initiatives such as the US National Research Council’s (2012) *Framework for K-12 science education* and the associated Next Generation Science Standards, which proceed from the axiom that “science is a way of knowing” (National Research Council, 2013, 100) and thus focus narrowly on the places and practices that are most visibly and directly related to scientific knowing. A science curriculum that fully incorporates the STS and history of science literatures, in contrast, would emphasize that science is about much more than knowing. Science is a hybrid enterprise, entangled with commerce, governance, spirituality, family, politics, security, diplomacy, philanthropy, and so on. Even scientific knowing itself isn’t limited to a few epistemologically-intensive practices (reading, writing, designing experiments, debating results, etc.), but also builds on many practices that enjoy less epistemological privilege: filing bureaucratic paperwork; hiring, firing, and managing personnel; arranging travel; keeping the workplace clean and safe; ordering materials and equipment. So, while I salute the Nature of Science program’s emphasis on getting students to learn (and even emulate) what scientists *do* in aid of getting them to learn what (and *how*) scientists *know*, a curriculum based in the history of science and STS literatures would (1) include a much wider variety of things that scientists do; and (2) acknowledge that scientific knowing is a means to a much wider variety of ends, and rarely an end unto itself.^5^

## The Whole Curriculum in the Science Classroom, Science in All the Classrooms

The kind of curriculum I have in mind has some similarities with what Allchin (2013) calls “Whole Science.” In his view, students have too long been fed an abstract, idealized version of science—a diet of white bread, vitamin pills, and refined sugar, if you will. For a healthier relationship to science, Allchin argues, students should consume Whole Science—analogous to wholewheat bread or, more generally, “whole foods”—which includes embarrassing yet essential aspects of science such as error (Martin, 2004), tedium (Weichselbraun, 2016), fraud (Park, 2020; Reich, 2009), denialism (Oreskes & Conway, 2011), and the creation of ignorance (Proctor & Schiebinger, 2008) alongside the creation of robust knowledge. As historians have shown, those things are definitely part of science. Yet why stop there? A science pedagogy rooted in the history of science literature would indeed include a warts-and-all picture of scientific reasoning, yet it could also venture far beyond reasoning, and even beyond “science” as conventionally understood. Playing on Allchin’s nutritional analogy, we might say that feeding science students Whole Science isn’t going to make them “healthier” unless that dietary change is accompanied by *lifestyle* changes—the pedagogical equivalent of moderate exercise, pro-social interaction, and avoiding drugs and alcohol—that span the Whole Curriculum. Hence the motto of my proposal: the whole curriculum in the science classroom, scientific practice in all the classrooms.

To understand what I mean by Whole Curriculum, think again about all the different things scientists do all day. Some of scientists’ quotidian activities could be flagged in *all* the educational situations that students encounter, not just the ones that are primarily oriented to science: hence, “scientific practice in all the classrooms.” At the same time, scientists’ many and varied practices could also be incorporated into science education, even those practices that don’t appear on the surface to be “scientific:” hence “the whole curriculum in the science classroom.”

Limitations of space, time, and expertise mean I can’t map out an entire curriculum to go with this motto, but I can offer a few examples. Let’s start with reading. We know that good scientists read, both extensively and critically. They also read both for work and for leisure, i.e., both “the literature” and just plain literature (Dillon, 2018). Literary and STS scholars as well as historians have shown that scientists consume, write, and contribute to works of fiction, such as films and plays (Kirby, 2011; Labinger, 2023; York, 2015) as well—again, both for pleasure and as an integral part of their science.

Both critical and pleasurable reading and writing could be taught in the science classroom—and they would be *the same* thing that scientists do, not a sandbox simulation. True, the Next Generation Science Standards do contain elements related to “domain-specific” reading and writing, but they don’t acknowledge the frequent entanglements of reading and writing for work *and* leisure, nor the significant overlap and cross-pollination between scientific texts and works of fiction and literature. Yet, there’s a large body of scholarship demonstrating those entanglements; and the scholars conducting that research even have their own professional society, the Society for Literature, Science, and the Arts.

Of course, critical and pleasurable reading are (hopefully) also taught in other classrooms—but there, reading isn’t usually flagged as something that *scientists* do. Yet, if we could teach reading as a scientific practice both in the science classroom and in the literature classroom, all kinds of benefits could transpire. Both critical and pleasurable reading are good skills for all students *qua* learners: becoming such a reader is an important component of *Bildung*. Inevitably, some students like the science classroom but not the literature classroom; by inserting a bit of scientific reading and writing in both classrooms, those students might have a more positive experience of critical and pleasurable reading. For such students, this approach also offers some forewarning that a career in science entails a lot of reading; better they find that out sooner rather than later. Conversely, for students who like the literature classroom but not the science classroom, teaching scientific reading in the former might make the latter more appealing. It’s good for science, and for society more generally, if people who start out by liking reading more than science nevertheless end up in the scientific workforce.

It’s also good for society if students learn early on how to be critical and pleasurable readers *of science*—i.e., to want to read about science, but also to know how to critically navigate a chaotic information environment and find trustworthy sources of science to read. Finally, I suspect—though this would need further study—that it’s to both science and wider society’s benefit if students see from early on that good scientists enjoy reading critically, but also that scientists put a lot of *hard work* into their reading: i.e., that “doing your own research” isn’t a matter of watching a couple YouTube videos, but rather that it is a disciplined practice that wears scientists down, physically and mentally, as much as it lifts them up.

So much for reading; for my second example let’s consider mathematics. Even more than science education, math pedagogy is geared toward training a science and engineering workforce that is imagined as needing statistics, trigonometry, and calculus on a daily basis. Relatedly, science curricula such as the NGSS present mathematization (i.e., the conversion of research questions into a calculable form) and mathematical analysis as *essential* features of all of science. Yet, all fields that are presently math-intensive underwent a—usually hotly contested—process of mathematization in the past (Gingras, 2001; Porter, 1995; Shapin, 2022; Siegmund-Schultze, 2018); and even today there are plenty of scientists who don’t actually use that much math. It’s rather unscientific—and certainly not consonant with what historians have shown—to *require* scientists to see the world in mathematical terms. Part of a Whole Curriculum perspective, then, would teach students to first ask whether their research question *should* be mathematized, and what the benefits *and costs* of doing so might be.

Moreover, even those scientists who use math every day often don’t need high-powered math. To quote Virgil Elings (1981) again, “it isn’t true that one needs a course in differential equations or Fourier analysis in order to design top-notch instruments.” Elings’ approach was rather to teach students how to figure out what math they needed for a specific project and how to then learn that math quickly on their own. What’s more, the math that many scientists need every day isn’t even scientific math, at least not in any distinctive sense. Many scientists spend their time reading and composing budgets, calculating the depreciating value of their equipment, dealing with loans, taxes, payrolls, insurance, student grades, and other applied arithmetic. That might sound mundane, yet scientists’ finances have been shown to have significant economic, scientific, and technological consequences (Castelnovo et al., 2018; Bianchini et al., 2019).

Now, I’m not proposing that we stop teaching math and science students calculus. Instead, I’m advocating for the teaching of a small amount of practical math—a kind of math that many scientists use every single day—in the science classroom and in the mathematics classroom, and for the designation of that kind of math *as scientific* in both classrooms. That kind of math would indeed be useful to future scientists; again, for those who already think they want to pursue a career in science, it’s best that they learn early on that part of such a career could well entail reading budgets and meeting payrolls. Learning some practical math would also be useful to ordinary citizens, whether they go on to a career in science or not. Moreover, teaching practical scientific math in both the math and science classroom conveys something important about what contemporary science *is*—i.e., science today is not the lone genius having a “eureka” moment, but is rather a vast hive of economic and bureaucratic activity that requires complicated bookkeeping.

That’s math; now, maybe even more controversially, let’s think about how to bring cookery into the science classroom. It’s trite but true to say that cooking is chemistry and *vice versa*; historically, there has indeed been much interplay between laboratory and kitchen (Gooday, 2008; Werrett, 2021). Yet, if students are exposed to cookery at school, it is probably not in the science classroom. In my junior high school—in a progressive Midwestern college town in the late 1980s—I was taught some cooking basics, but within the framework of “home economics” education. Those basics weren’t presented as having much to do with science, yet in my experience, they were quite handy once I started doing laboratory assignments in high school and college chemistry courses. They also proved handy once I moved into my own apartment and had to feed myself every day.

Thus, teaching cookery in the science classroom could help students better understand chemical concepts and practices. I suspect it could have other benefits as well, such as broadening the appeal of science by showing the applicability of science to something we all do every day (consuming food and drink). For students who have some experience in the kitchen, connecting science and cookery could be a way to activate their prior knowledge. Maybe it could broaden the appeal of—and respect for—cookery among science-oriented students who might otherwise be attracted to a Silicon Valley brogrammer conception of scientists as disembodied brains-in-vats fueled only by Soylent Green. Finally, everyone just should know how to feed themselves and how much work goes into feeding others; not all schools have a home economics classroom where that knowledge is taught, but most schools have a science classroom where it *could* be taught.

More common—though unfortunately dwindling—are schools with art classrooms, or at least art teachers. As noted earlier in this article, the intersections of art and science are many, longstanding, and mutually beneficial. That point isn’t completely obscured by NOS-oriented curricula; the Next Generation Science Standards do say, for example, that “creativity and imagination are important to science.” Yet, such curricula don’t really convey that scientists have aesthetic sensibilities, they apply those sensibilities in their science (Lee, 2020), and that science and aesthetics are mutually produced. Dance (Myers, 2012), musical (Supper, 2013) and visual (Lynch & Edgerton, 1996) literacy, artisanal knowledge (Onaga & Douny, 2023)—these aren’t important to *all* scientists, but neither is mathematics; and historians and ethnographers have shown over and over that aesthetic pursuits are important to *some*, even many, scientists. If we teach aesthetics in the science classroom, we are teaching a scientific practice. If we take some time in the art classroom to acknowledge that scientists apply their aesthetic sensibilities, then we might make art more appealing to students who like science and science more appealing to students who like art.

One could even lever science pedagogy into the gymnasium and athletic field, or sports and exercise into the science classroom. As a number of historians have shown, physical activity has been intimately tied to scientific research in many times and places (Hevly, 1996; Owens, 1985; Warwick, 1998). Again, all students—*qua* students and *qua* citizens—could benefit from seeing that the life of the body and the life of the mind have a close historical connection. Especially students who plan to continue in science should know from early on that science is hard on the body. Conversely, students who think they like sports and exercise but don’t think they like science should know that there’s no inherent opposition between the two, and the two *can* be mutually reinforcing.

## Words of Hope and Caution

Critical readers might at this point have a number of objections to my sketch of a Whole Curriculum rooted in the history of science literature. For instance, it’s certainly fair to complain that my program would partially capture non-science classrooms for the benefit of science education. My response is that Whole Curriculum thinking puts as much art, cookery, literature, etc. into the science classroom as it puts science in those other classrooms. I’ve tried to indicate the ways that that trade would have mutual benefits for the science educator, other educators, students who continue in science, students who don’t, and for society more generally.

Others may object that the Whole Curriculum program reimagines, well, the whole curriculum, and that it is therefore infeasible if it is implemented as a ground-up strategy, and totalitarian if it is implemented as a top-down plan of action. I don’t think there’s much danger that it will be imposed from above anytime soon, though I certainly wouldn’t mind if some national authority (in whatever nation) were to gesture to it in future curriculum reforms. The Whole Curriculum idea doesn’t have to entail a totalizing revamping of the curriculum, though. What I have in mind wouldn’t need to displace more than a small fraction of the current curriculum, both in the science classroom and in all the other classrooms—a day for art and science here, a day for science and cookery there, presented as a break from the mandated curricular fare, would suffice. Those kinds of changes also wouldn’t need to be instituted from top-down. One could imagine, for instance, a school where the chemistry teacher and the art teacher start having coffee once a month, just to learn from each other, until eventually the art teacher starts making occasional appearances in the chemistry classroom and the chemistry teacher in the art classroom in order to guide students through a couple of hours discussion of, say, the aesthetics of molecular models.

Finally, some readers will object that I’ve reduced science to banalities (eating, sleeping, budgets, etc.) and that I’ve thus neglected what makes science special. I am, certainly, arguing that science educators and frameworks such as the Next Generation Science Standards shouldn’t set “scientific” reasoning, knowledge, and practices apart from ordinary reasoning, knowledge, and practices—science bleeds into other walks of life, and the things scientists do are also done by non-scientists. Yet, science doesn’t need to be unique to be special. Science is a human achievement, not a superhuman achievement; and that makes it all the more remarkable that humans have achieved so much using practices that science shares with other—decidedly human—walks of life. The Whole Curriculum doesn’t trivialize science; rather, it helps students understand that all their classrooms have both extrinsic and intrinsic worth, that the knowledge gained in every classroom can generate both wonder and instrumental gain, and thus that all the classrooms are worthy of mutual respect.

Mutual respect could, I hope, in turn lead to that much-desired end of contemporary science education: namely, critical and justified *trust* in science. As Allchin (2022) notes, the problem is not actually a matter of trust, *per se*:

analysis of the science contrarians indicates that they do, indeed, trust science… Unfortunately, they trust the *wrong* science. Or, perhaps more appropriately, their sources of scientific information do not report scientific consensus. They trust the wrong *voice* for science. To address the question of scientific misinformation, then, educators must shift their focus from *what* to trust to *who* to trust.

Both for students who won’t go on to careers in science and for those who will, showing that science is many things, and shares much in common with non-scientific pursuits, should yield a generation of citizens who better understand what scientists do and how scientists establish claims that are as robust as the claims made by any other profession. Teaching cookery in the science classroom invites comparison between the scientist and the professional chef: amateur chefs can cook a good meal, professional chefs a bad one, but we go to restaurants in part because we trust that, more likely than not, the trained chef can make the dishes on their menu better than we could ourselves. Teaching science in gym class invites comparison between science and athletics: widespread amateur participation is a good thing whether in science or athletics, and amateurs can spur innovation whether in athletics (Franke & Shah, 2003) or science, and yet it is surely uncontroversial to expect the professional athlete to, in general, outcompete the amateur.

Likewise, the Whole Curriculum approach can convey that non-professionals can make valid discoveries, and professional scientists can make questionable claims; but we consult the professional scientist on many weighty matters because on balance scientists’ trained judgment leads them to reliable insights. The Whole Curriculum approach conveys that science is not an ivory tower, remote both from real people and the real world, but is in constant contact with all walks of life—for better and for worse. The Whole Curriculum approach conveys that scientific knowledge is not gained easily, by, for example, consuming social media, but is hard work—physical, mental, sometimes pleasurable, and, most importantly, *collective* labor—that relies on social interactions, complex organizations, and thus on interpersonal and administrative *nous*.

Above all, the Whole Curriculum conveys that science education shouldn’t just happen in the science classroom, because science doesn’t just happen in the lab (and a handful of other canonical sites). Relatedly, science doesn’t just happen in the Global North, as the other articles in this special issue explore further. The diversity of places where science unfolds means that people from all countries and many walks of life have the capacity to actively learn from, to participate in, and to contribute to science in one way or another. Science educators have an important role to play in affirming and helping students and citizens to recognize that capacity. Yet, doing so requires going beyond a narrow understanding of what scientists do and *where* they do it.

## Data Availability

All references in the manuscript are to published documents or to unpublished sources that are available in public archives at the cited location.

## Footnotes

1 Again, readers can consult the references in Mody (2022), especially for archival documents charting Elings’ and Phillips’ careers. The specific claim about Croiset is also documented in a published source, Oltmans (2004).

2 Here, and in the following, readers can reference Mody (2011) for information about Digital Instruments.

3 See the items in Box 19, DP (Departmental) series, Folder 12 Atomic Energy Commission (1949–1951), Robert S. Kerr Papers, Carl Albert Center, University of Oklahoma, especially a letter to Senator Kerr from Waldo E. Cohn, October 9, 1951. Note that Cohn signed the letter “Principal Biochemist, Oak Ridge National Laboratory/Conductor, Oak Ridge Symphony Orchestra.”

4 During ethnographic fieldwork at materials science conferences in the early 2000s I witnessed laboratory experiments being performed in instrument manufacturers’ booths at the conference vendors’ exhibit—experiments on novel materials brought to the exhibit by potential customers (i.e., the experiments weren’t staged but could, and did, deliver novel scientific insights). While probably not common, others have reported similar novel experiments performed at conferences. See monitor report, Gordon Research Conference on Nucleic Acids (1971), in box 127, binder for year 1971, Records of the Gordon Research Conferences (2001–016-001), Science History Institute Archives. The monitor records that “one speaker indicated that he had completed the *experiment* in his room at the Conference” (emphasis in original).

5 I need to be careful not to broach confidentiality, but suffice to say that my arguments in this essay (and especially those in this paragraph) were greatly influenced by a manuscript I was asked to peer review which, to my knowledge, has not yet appeared in print anywhere. As I don’t know the author’s name, I can’t cite them, but let this footnote stand as an invitation for authors who think I may be gesturing to their ideas in this paragraph to get in touch with me.
